# Supplementary figures and images for: Genetic Distribution of Five Spinocerebellar Ataxia Microsatellite Loci in Mexican Native American Populations and Its Impact on Contemporary Mestizo Populations
Source: Genes (Basel). 2022 Jan 16;13(1):157. doi: 10.3390/genes13010157 (PMC8775409; doi:10.3390/genes13010157)

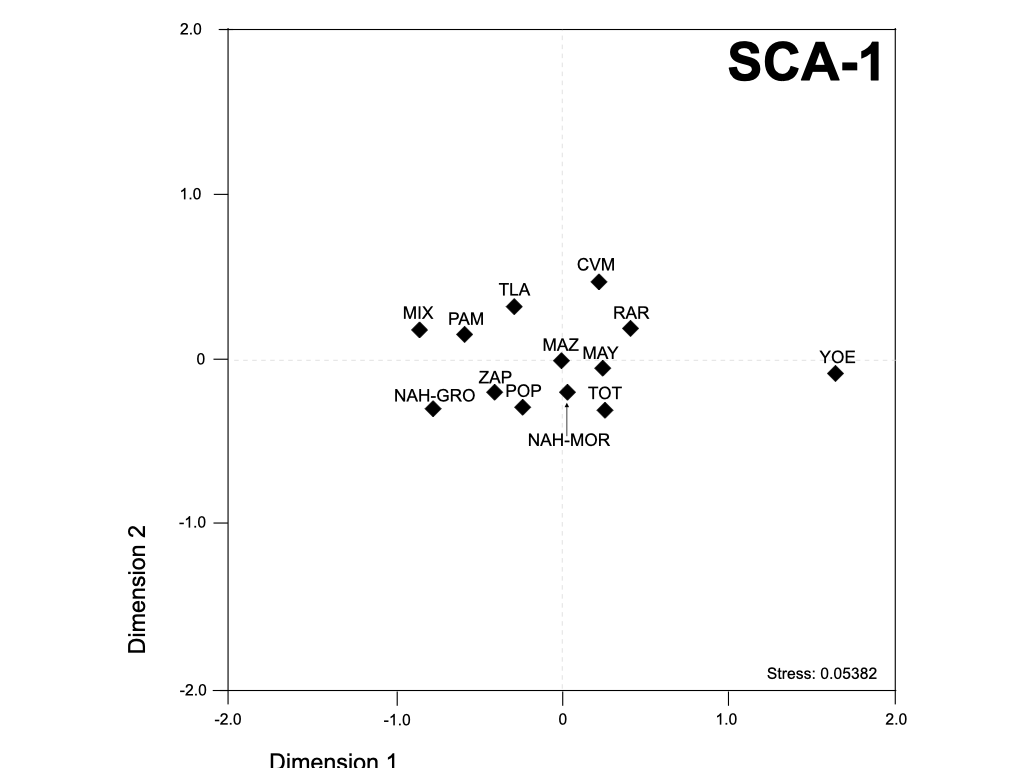

Supplement: Supplementary file 1 [file genes-13-00157-s001.zip › Supplementary materials Gomez R/Figure S3.tiff]

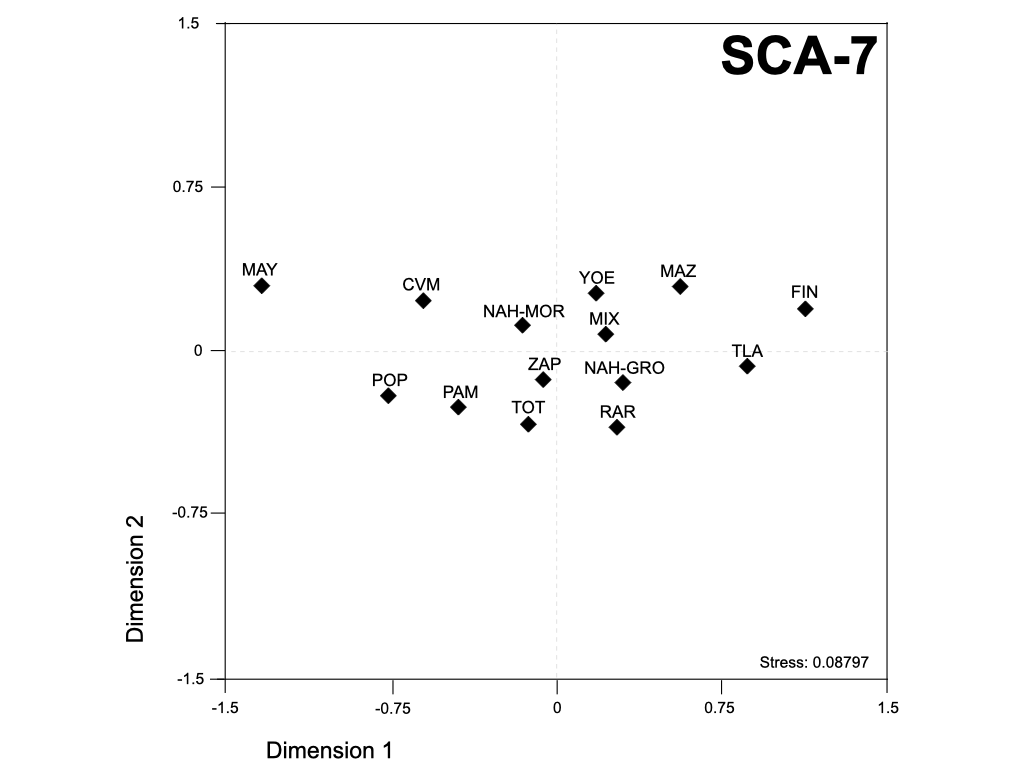

Supplement: Supplementary file 1 [file genes-13-00157-s001.zip › Supplementary materials Gomez R/Figure S11.tiff]

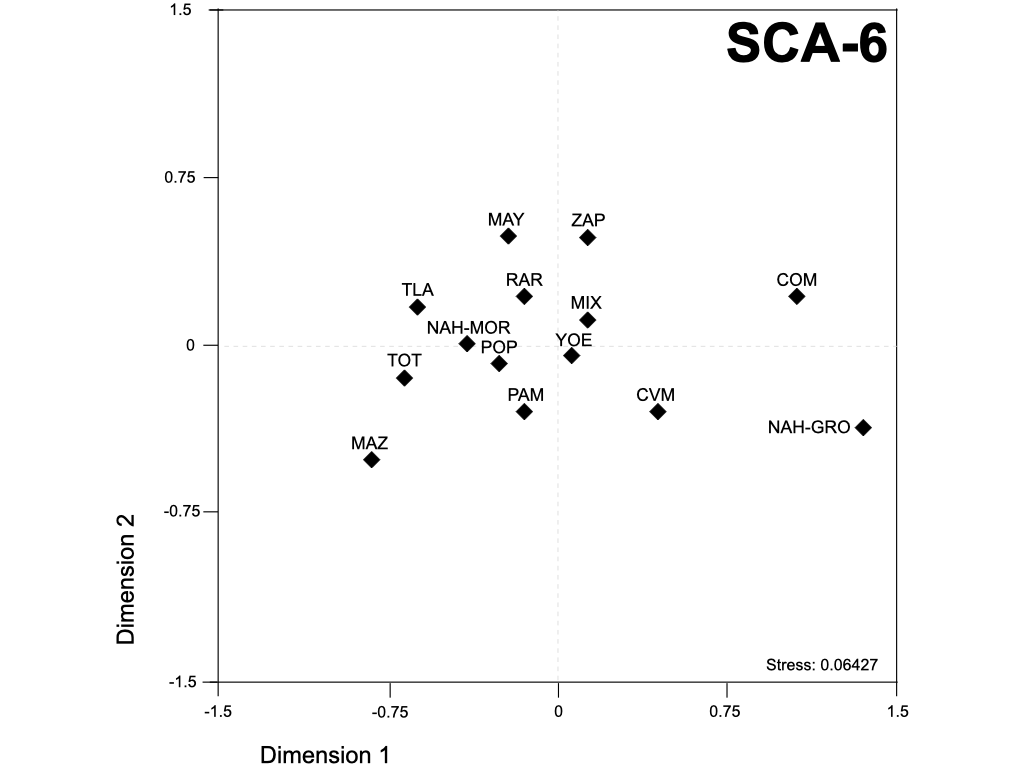

Supplement: Supplementary file 1 [file genes-13-00157-s001.zip › Supplementary materials Gomez R/Figure S10.tiff]

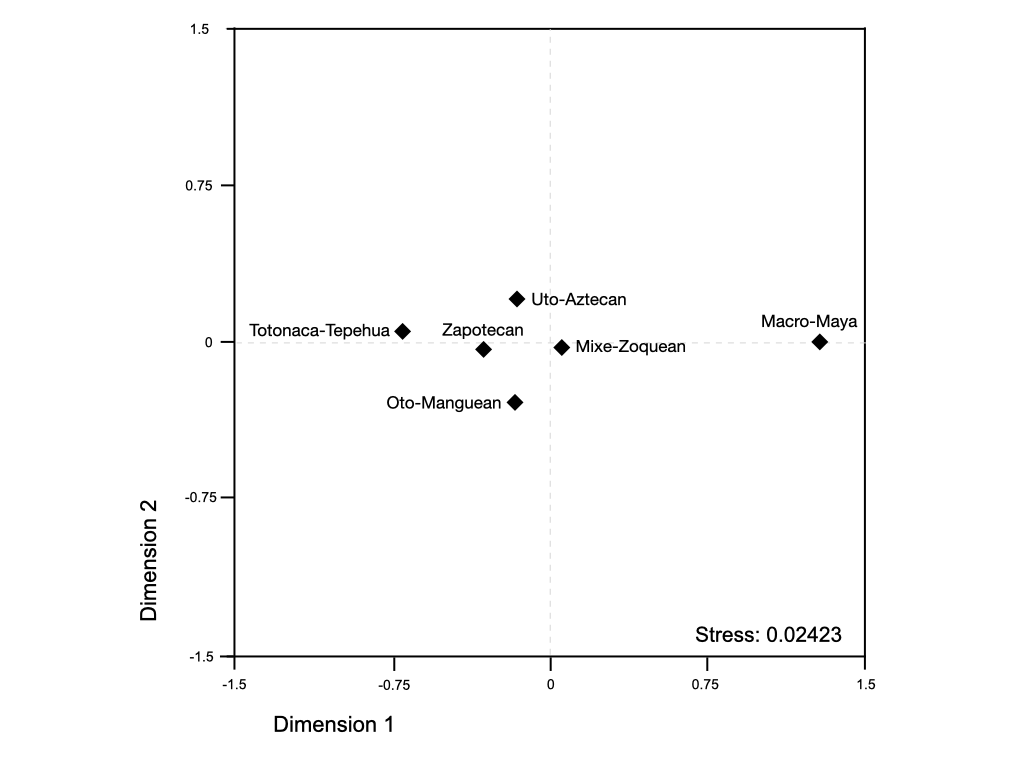

Supplement: Supplementary file 1 [file genes-13-00157-s001.zip › Supplementary materials Gomez R/Figure S2.tiff]

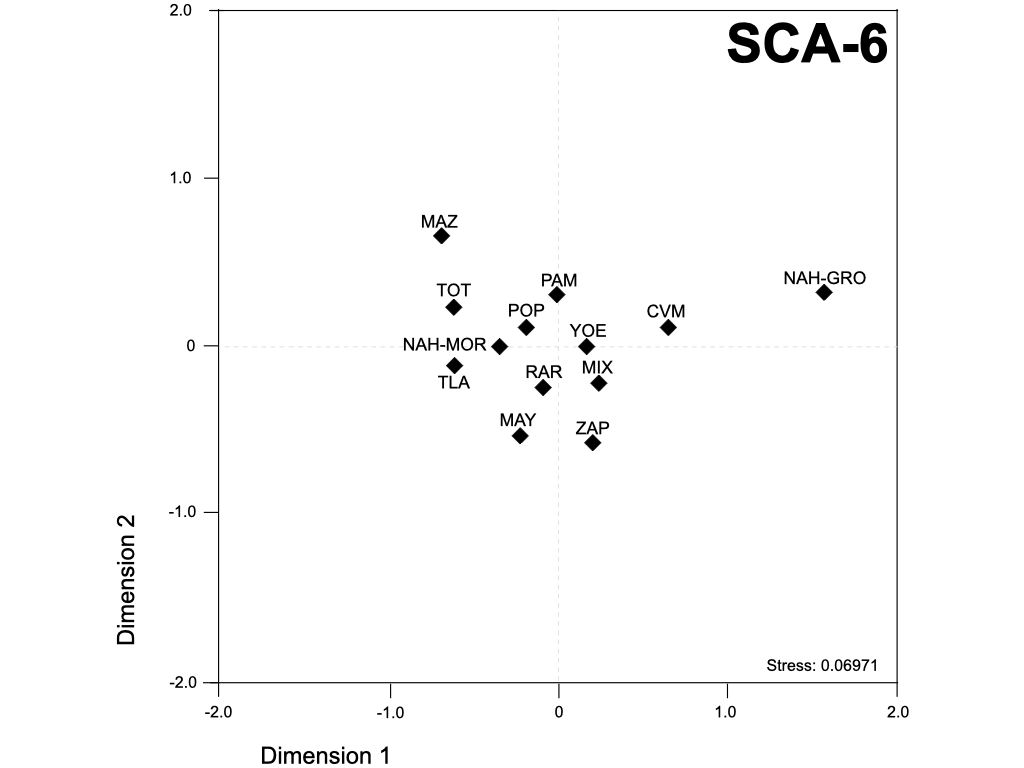

Supplement: Supplementary file 1 [file genes-13-00157-s001.zip › Supplementary materials Gomez R/Figure S5.tiff]

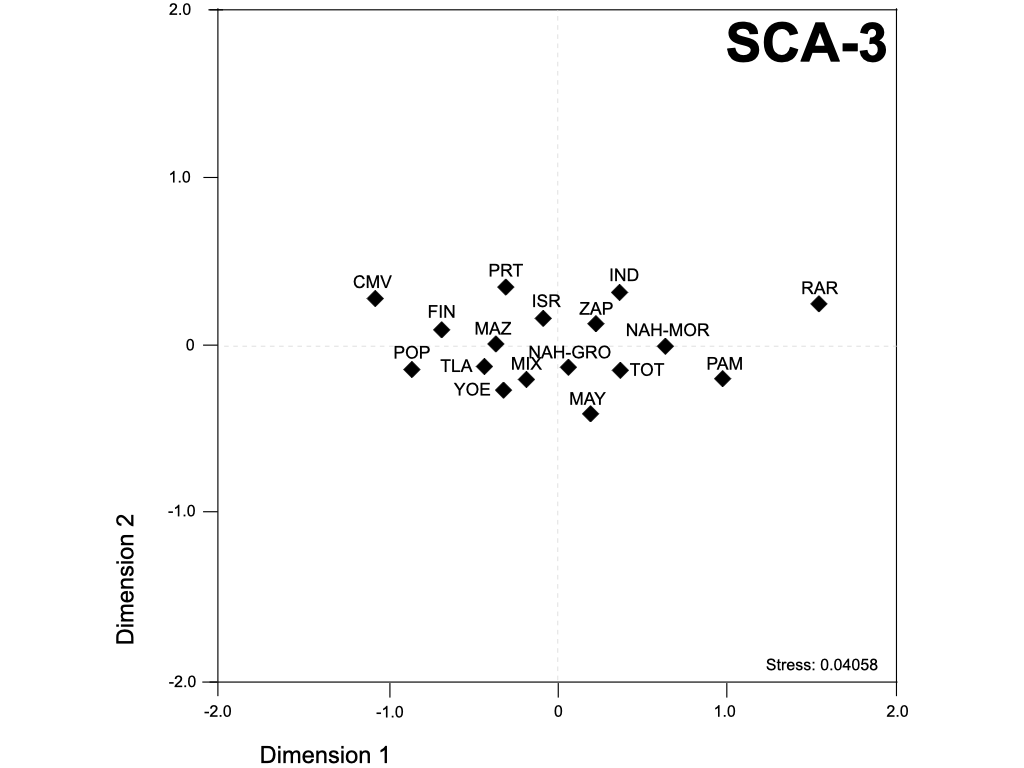

Supplement: Supplementary file 1 [file genes-13-00157-s001.zip › Supplementary materials Gomez R/Figure S9.tiff]

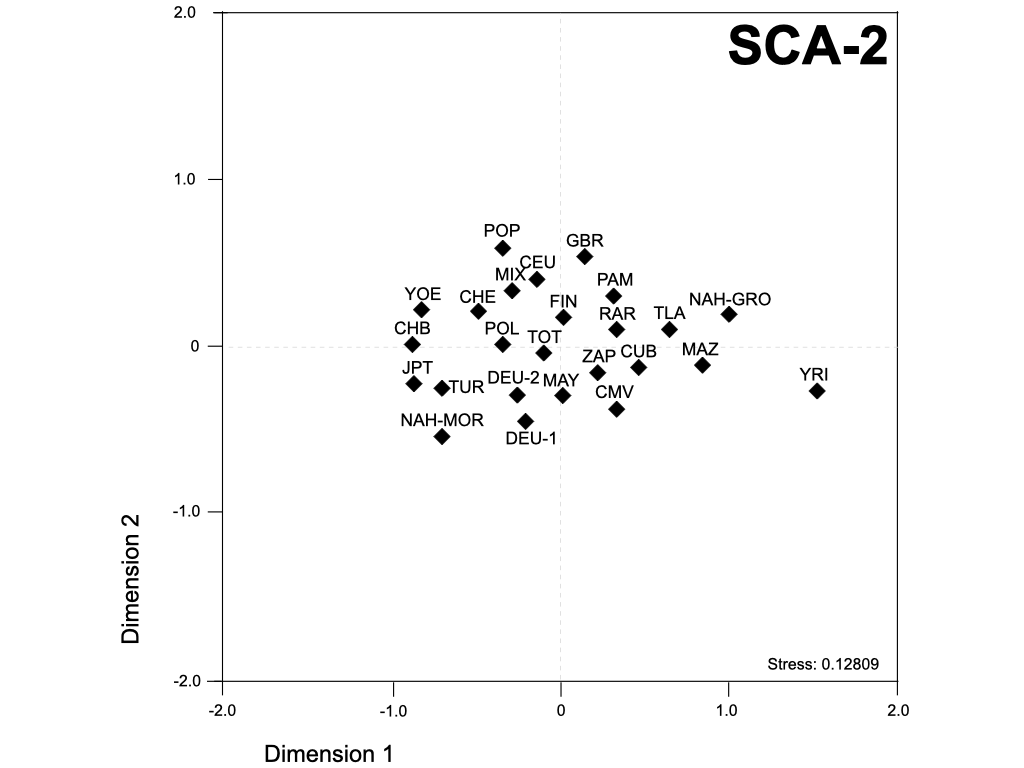

Supplement: Supplementary file 1 [file genes-13-00157-s001.zip › Supplementary materials Gomez R/Figure S8.tiff]

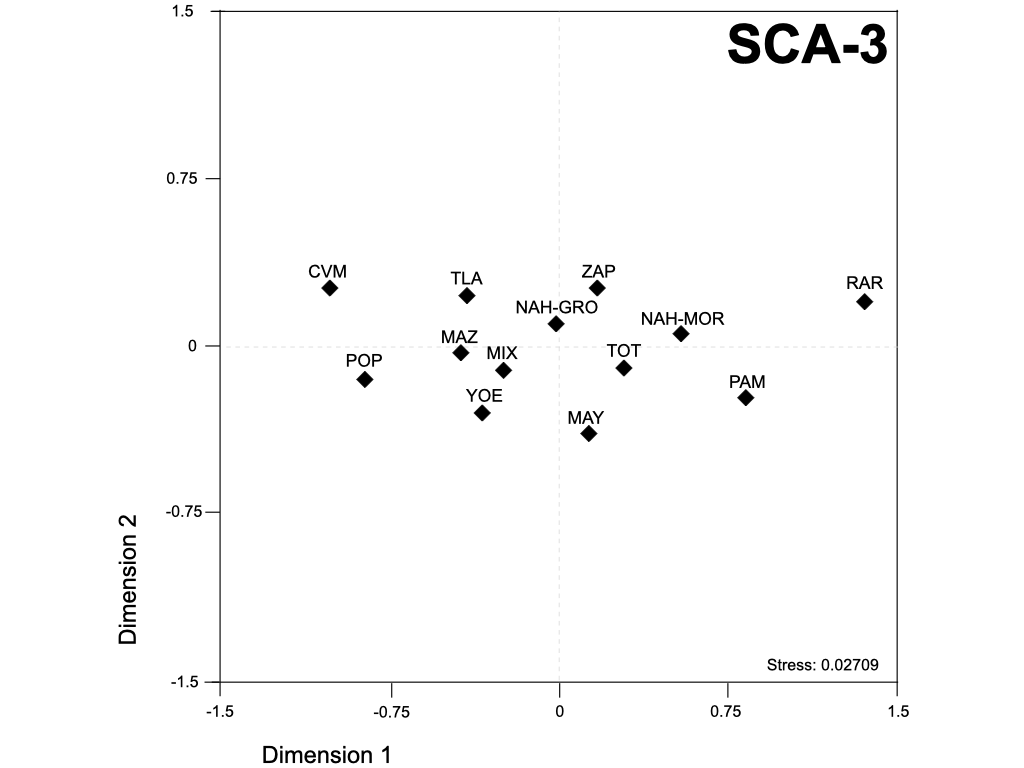

Supplement: Supplementary file 1 [file genes-13-00157-s001.zip › Supplementary materials Gomez R/Figure S4.tiff]

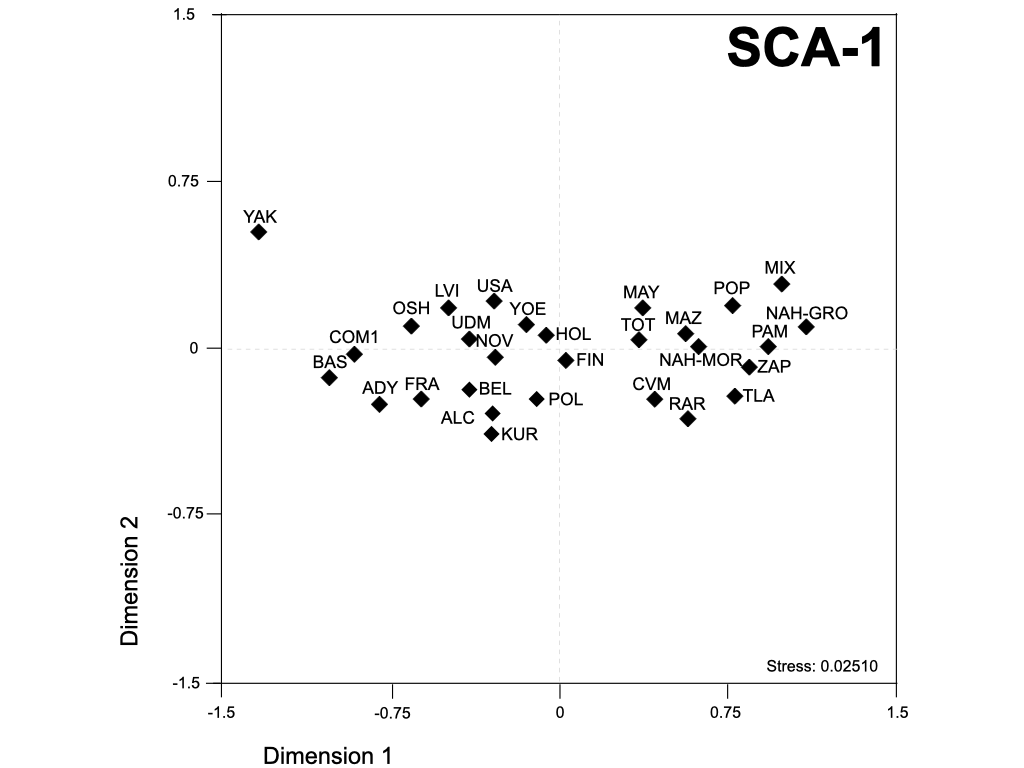

Supplement: Supplementary file 1 [file genes-13-00157-s001.zip › Supplementary materials Gomez R/Figure S7.tiff]

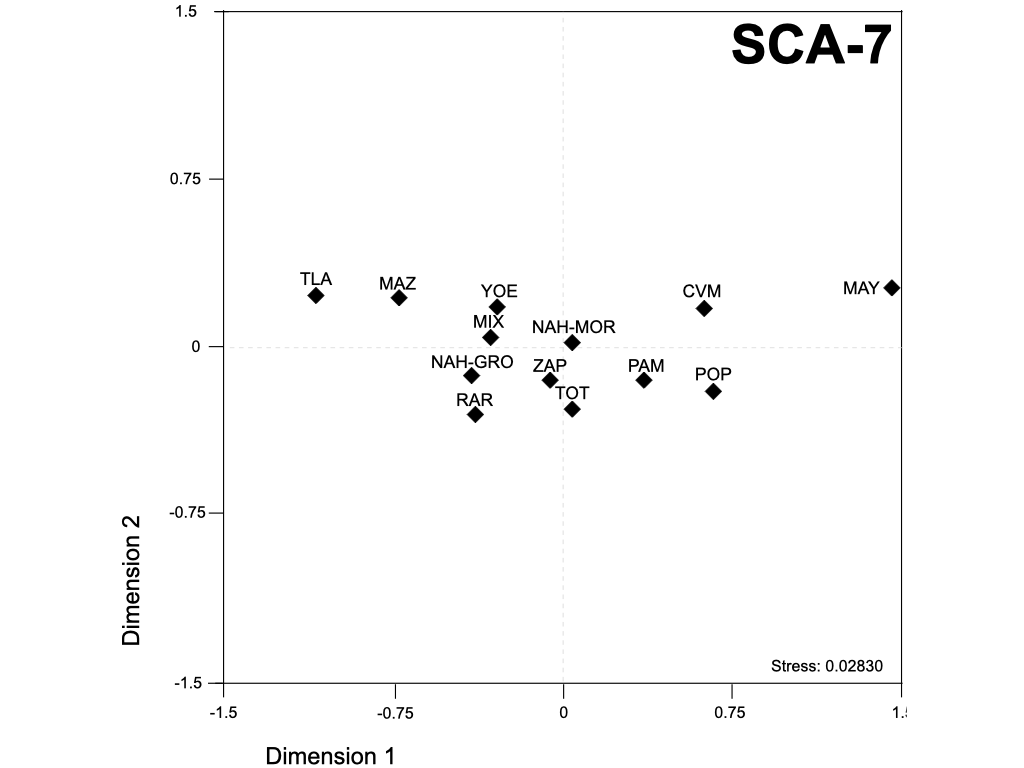

Supplement: Supplementary file 1 [file genes-13-00157-s001.zip › Supplementary materials Gomez R/Figure S6.tiff]

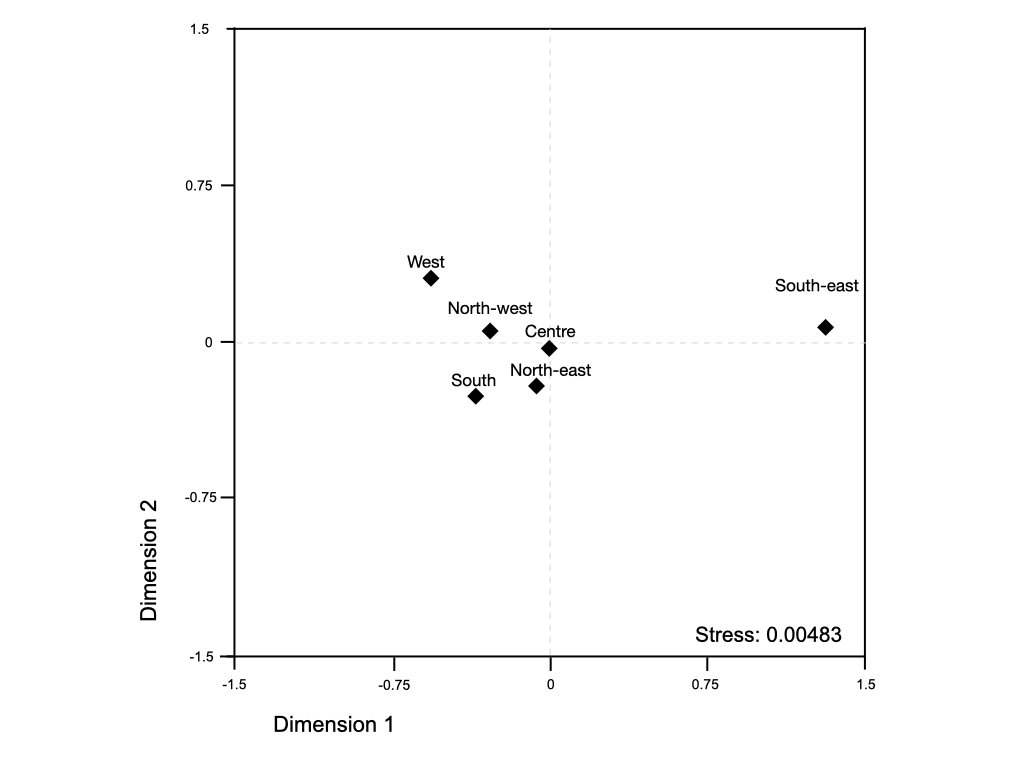

Supplement: Supplementary file 1 [file genes-13-00157-s001.zip › Supplementary materials Gomez R/Figure S1.tiff]
